# Give science and peace a chance: Speeches by Nobel laureates in the sciences, 1901-2018

**DOI:** 10.1371/journal.pone.0223505

**Published:** 2019-10-08

**Authors:** Massimiano Bucchi, Enzo Loner, Eliana Fattorini

**Affiliations:** Department of Sociology and Social Research, University of Trento, Trento, Italy; Universidade de Brasilia, BRAZIL

## Abstract

The paper presents the results of a quantitative analysis of speeches by Nobel laureates in the sciences (Physics, Chemistry, Medicine) at the Prize gala dinner throughout the whole history of the Prize, 1901–2018. The results outline key themes and historical trends. A dominant theme, common to most speeches, is the exaltation of science as a profession by the laureate. Since the 1970s, especially in chemistry, this element becomes more domain-specific and less related to science in general. One could speculate whether this happens chiefly in chemistry because its area of activity has been perceived to be at risk of erosion from competing fields (e.g. physics, biology). Over time, speeches become more technical, less ceremonial and more lecture-oriented. Emphasis on broad, beneficial impact of science for humanity and mankind (as emphasised in Nobel’s will) is more present in laureates’ speeches during the first half of the XXth century, while its relevance clearly declines during the last decades. Politics and its relationship with science is also a relevant topic in Nobel speeches. Particularly between the two World Wars, science is seen as terrain where nationalistic stances and fights among nations could actually find a context for peaceful competition and even cooperation.

## Introduction

Studying the Nobel Prize in the sciences is an extraordinary gateway for understanding transformations in the public image of science–and of scientists–from the early XXth century to the present and also understanding how the Prize itself contributed to shaping that image. The Nobel Prize announcements are in fact one of the occasions when science makes global headline news in the media; the halo and reputation of the Prize reaching even those audiences which are not much interested in science; in fiction–from Hollywood movies to the Simpsons–‘Nobel’ has become a metonym for brilliant minds, genius and successful science (for a more elaborate version of this argument and a more general analysis of the role of the Nobel Prize with regard to the public image of science in the XXth century, see [1, 2, 3]).

As Harriet Zuckerman [4] noted in her book Scientific Elite,

I am inclined to think that the principal effect of the prize on science in the large is indirect; its influence on the public’s image of science probably counts for more than its function as incentive for scientific accomplishment. Decades-long reiterated attention to the prizes and the laureates in the public press, to their great achievements and to the ceremony honouring them, announces to the public that great things are stirring in science, things worthy of public admiration and public support (p. xxviii).

In this context, public speeches by laureates during the Nobel festivities bear significant interest, in particular speeches given at the Nobel banquet. While public lectures are more focused on the technical aspects of research, banquet speeches offer several insights into the images of science and scientific profession that Nobel scientists implicitly or explicitly convey to the audience [5].

Our study has analysed all of the 218 speeches given by laureates in physics, chemistry, and physiology or medicine during the official Nobel banquet of 10^th^ December, throughout the whole history of the Prize, from 1901 to 2018, transcribed and available in English on the Nobel foundation official website (www.nobelprize.org). Traditionally, only one laureate in each area gives a speech at the banquet (normally the oldest one). For various reasons, some laureates did not travel to collect their prize in Stockholm (e.g. for health reasons, during war times, or simply because unable to travel) and therefore their banquet speech is not available [1].

The results outline key themes and historical trends, also pointing out directions for further analysis of these data.

## Data and methods

Data analysed in this work have been downloaded from the Nobel Prize official website (https://www.nobelprize.org/). A dataset with the list of the laureates selected for this research is available at https://figshare.com/s/8a096f5b5ed70d4aa299. The analysis includes all the speeches given by laureates in physics, chemistry, and physiology or medicine during the official Nobel banquet of 10th December, throughout the whole history of the Prize, from 1901 to 2018, transcribed and available in English on the Nobel foundation official website. The final database consists of 218 Nobel speeches, whose distribution among the three fields presents minimal differences compared to the overall Nobel population (Table 1).

**Table 1:** Comparison between data about speeches and the Nobel population.

	Speeches		Nobel population		
Category	n.	%	n.	%	Diff.
Chemistry	67	30.7	181	29.8	+0.9
Physics	77	35.4	210	34.6	+0.8
Physiology or Medicine	74	33.9	216	35.6	-1.7
Tot.	218	100.0	607	100.0	

Data are also in line with the Nobel population in terms of gender: during the history of the Nobel Prize only 20 women have been awarded in the sciences (3.3% of the total); 8 speeches from women are available (3.7% of the total number of speeches).

Quantitative analysis has been conducted with package Quanteda [6] and statistical programme R 3.5.2 [7]. After having been downloaded from the Nobel Prize official website, speeches have been put into a corpus and prepared for quantitative text analysis [8, 9]. The initial phase has focused on data cleaning and the construction of the document-feature matrix (DFM), i.e. the representation of texts in matrix format, with rows representing text words and columns representing their features.

## Science as a profession and hard work. Protecting disciplinary boundaries

**Fig 1:**
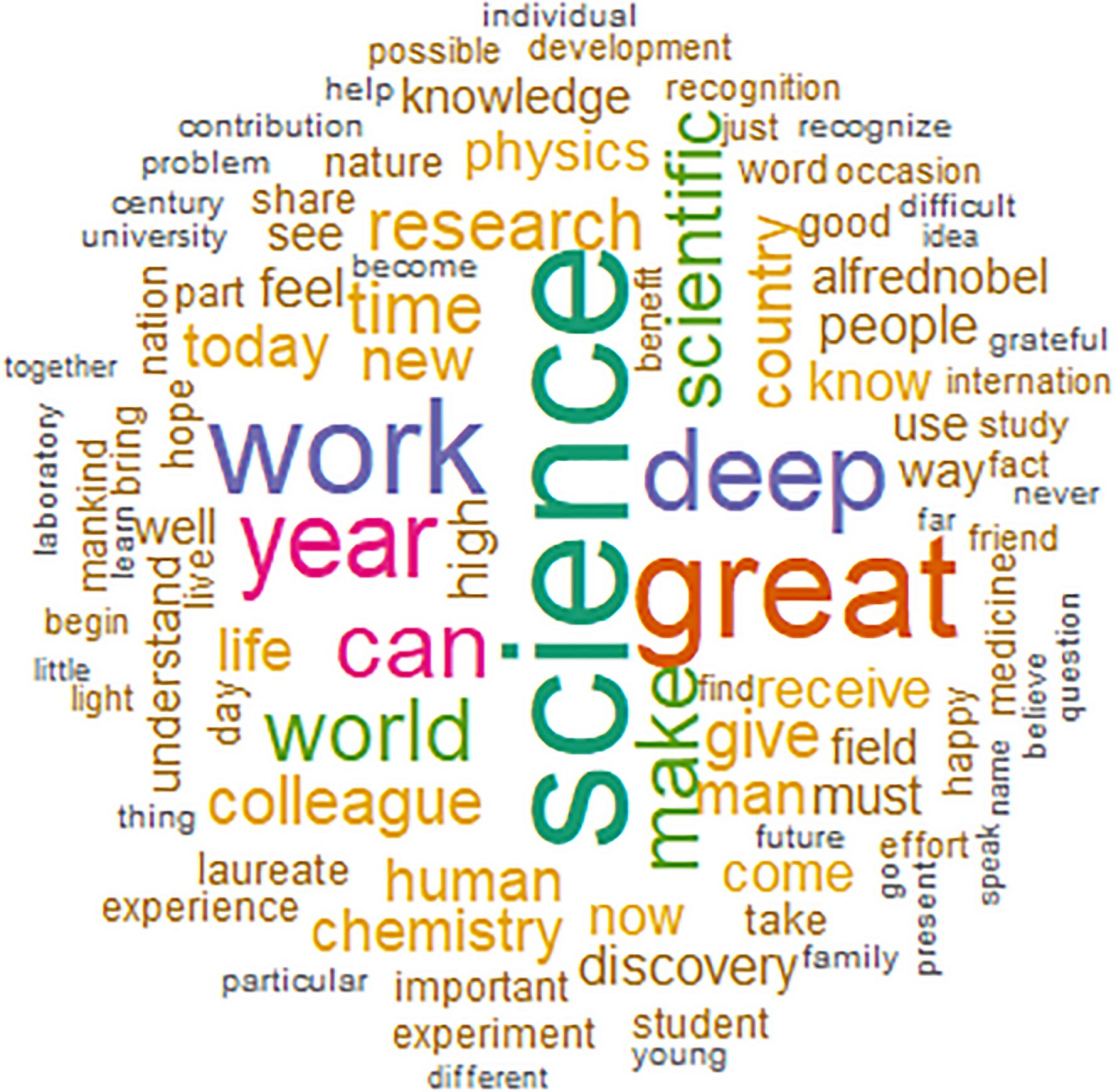
Word cloud of the most used words in Nobel banquet speeches.

A dominant theme, common to most speeches, is the exaltation of science as a profession by the laureate (Fig 1). An example of this theme is in the following quotation from Dante’s *Divina Commedia* by Carlo Rubbia (Nobel Prize in Physics, 1984):

This spirit of collective greed for discovery rather than for power and struggle is best described by Dante in the words of Ulysses sailing toward the limit of the earth, the Pillars of Hercules: (“for brutish ignorance your mettle was not made; you were made men to follow after knowledge and excellence”).

Science is often described as a profession requiring great dedication and sacrifice. The term “work” is widely used to describe the commitment by researchers.

In the words of Shuji Nakamura (Nobel Prize in Physics, 2014):

If I can tell you a little story of encouragement … when we began work on the blue LED in the 1980s, we were told again and again that what we were trying to do was impossible. Still, we persevered, working hard for many hours and years to develop this new technology.

The image of science given in the speeches is often concrete and practical. For Nobel laureates, science is not so much about theorizing and abstract thinking but about “making things”, hard work and intense effort.

Beside science as a profession, the laureates often celebrate their own field. General references to science as a whole during speeches decline over time in favour of reference to and praise of one’s specific field.

**Fig 2:**
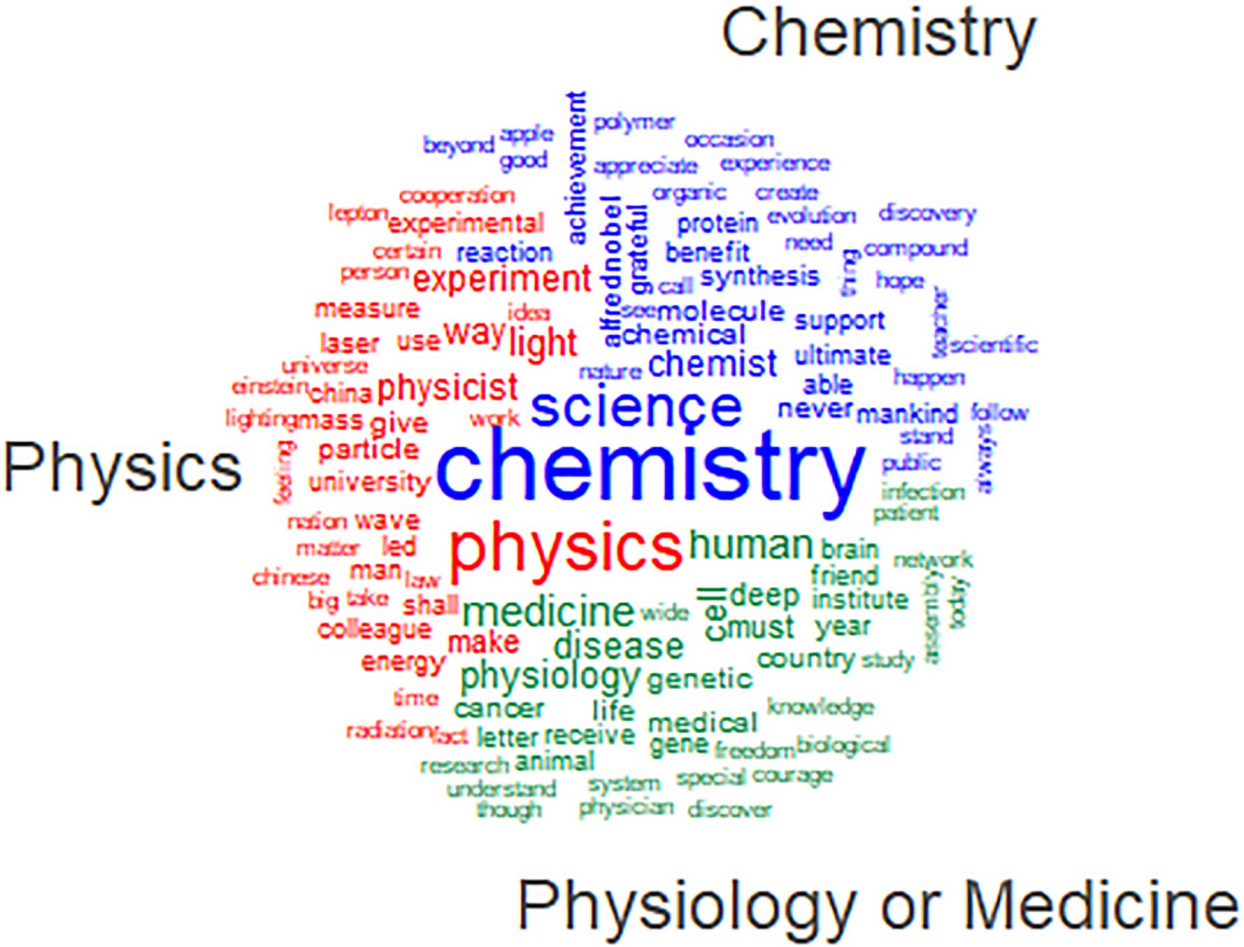
Nobel speeches by laureates in Physics, Chemistry and Physiology or Medicine: Word cloud comparison.

This is more common among chemists compared to physicists and, even more, medical researchers (Fig 2). A hypothesis to be further explored is that chemistry, during the last century, has repeatedly struggled to preserve its autonomy and distinctive boundaries vis-à-vis developments in other fields (particularly physics, but also biology). These developments have often been perceived by chemist as eroding their own specialization and intellectual ownership of relevant research themes [10, 11]. In terms of public visibility, long term studies of Nobel media coverage confirm that chemistry and chemists have been often shadowed by the other science fields involved in the Nobel Prize [12].

Extensive reference to one’s research field, however, can become self-referential and associated with more technical speeches over time [13, 14, 15]. This is what happens particularly from the Seventies and, once again, especially in chemistry. Technical terms and specific references to research themes–almost absent in early years–become more frequent in recent decades.

**Fig 3:**
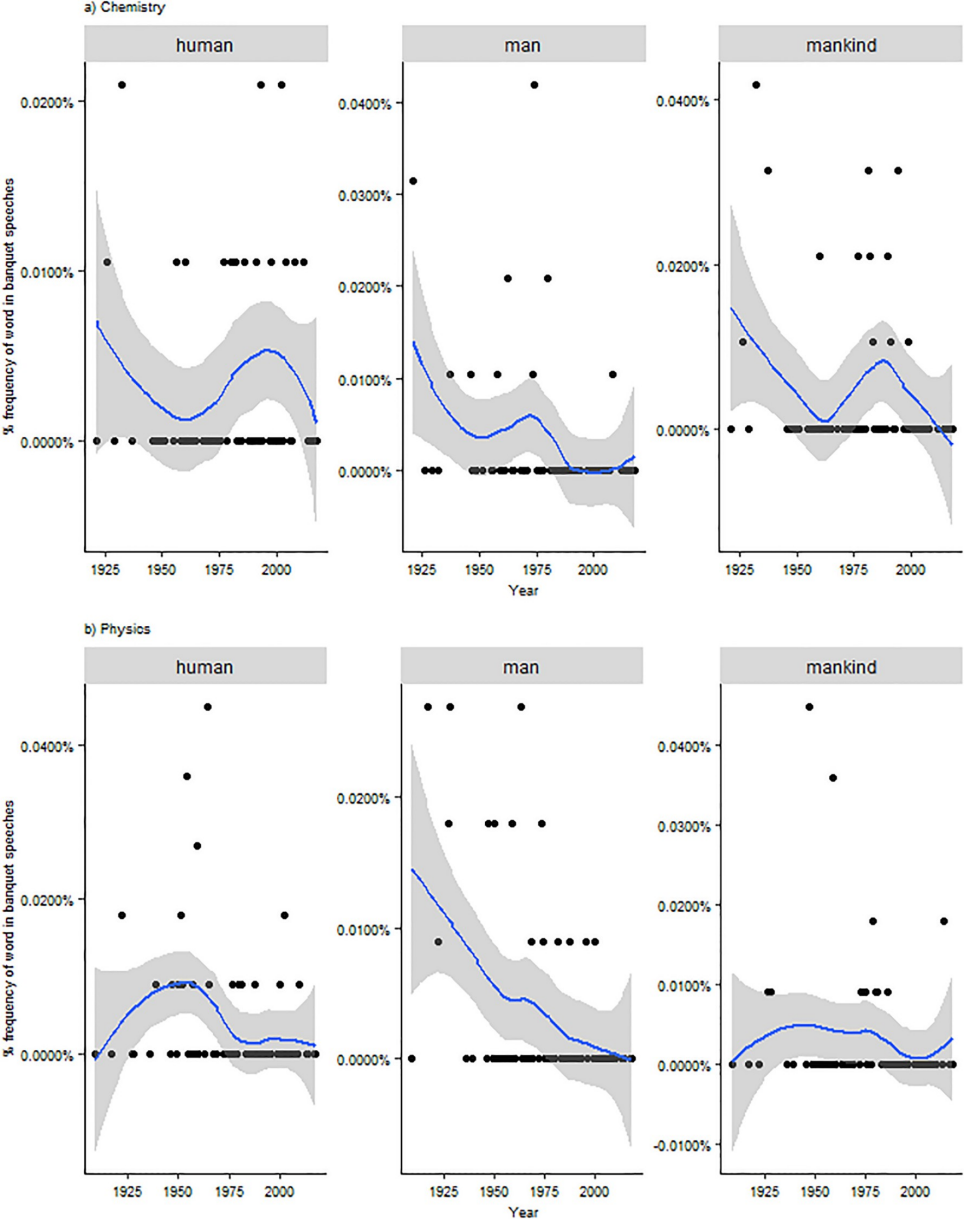
Mentions of “human”, “man”, and “mankind” across time, speeches by laureates in Chemistry and Physics. Dots represent the % of each term on the total of words for each year. The line represents the historical trend (confidence interval at 95%).

According to the original guidelines stated in the will of the founder Alfred Nobel, the Nobel Prize should reward those “who have conferred the greatest benefit to humankind” [16, 17]. This emphasis on broad, beneficial impact of science for humanity and mankind is more present in laureates’ speeches during the first half of the XXth century, while its relevance clearly declines during the last decades, particularly in physics and (more recently) chemistry (Fig 3).

**Fig 4:**
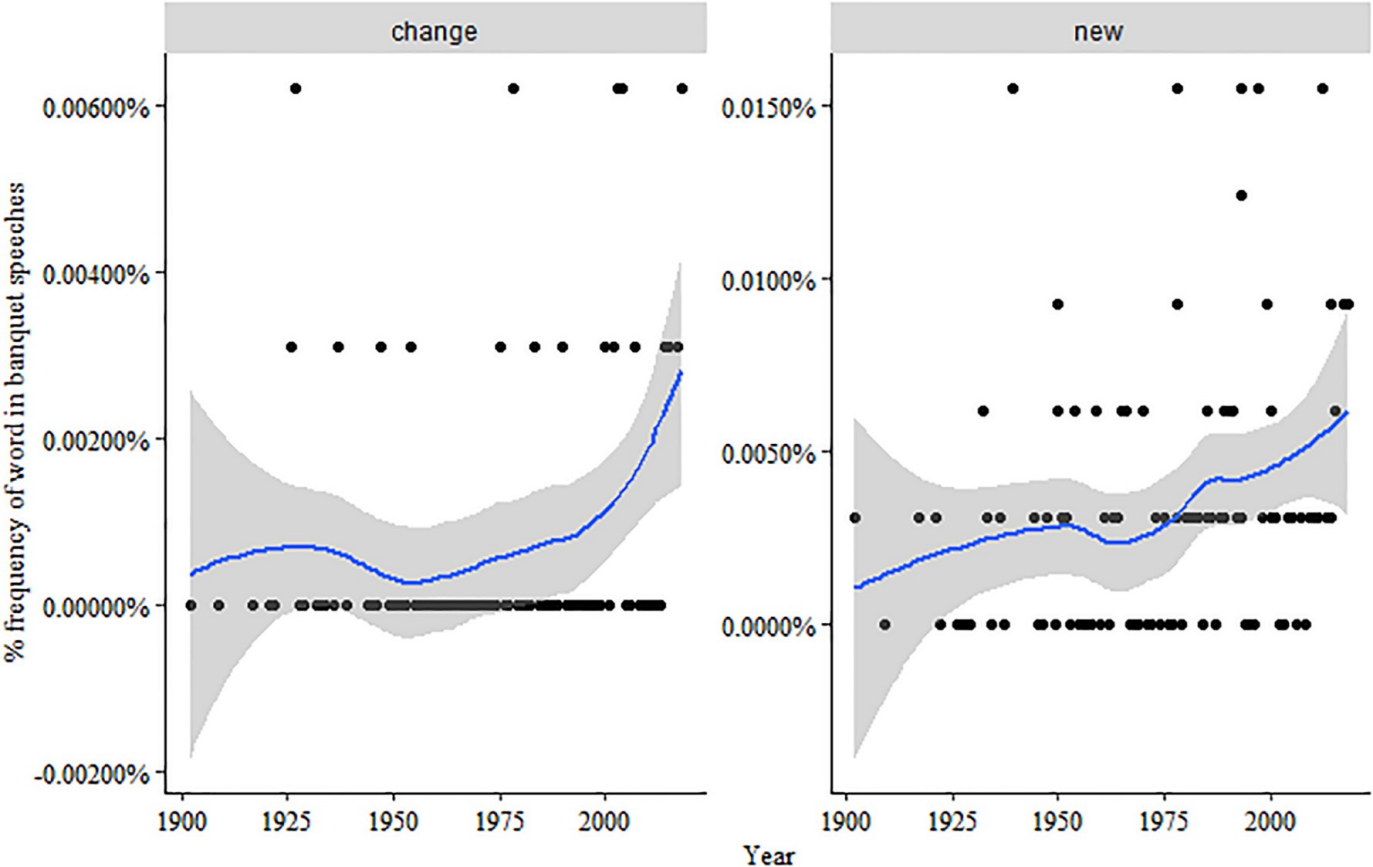
Mentions of “change” and “new” across time. Dots represent the % of each term on the total of words for each year. The line represents the historical trend (confidence interval at 95%).

This is matched by an increasing number of technical terms as well as by an emphasis on change and innovation that may reflect, particularly in the last two decades, an awareness of changes in the modes of knowledge production such as the increasing proximity to its context of application (Fig 4) [18, 19].

In short, banquet speeches are less frequently an occasion for grand visions and descriptions of the scientific enterprise, becoming more technical, less ceremonial and more lecture-oriented. The focus is increasingly on the detailed process which led to the specific discovery rewarded with the Prize, as in this speech by Tasuku Honjo (Physiology or Medicine, 2018):

The concept of cancer immunotherapy was theoretically proposed by the Australian Nobel Laureate Sir Frank Macfarlane Burnet over sixty years ago, and since then, a large number of people have tried to apply it, but without success. This was probably because their efforts focused on pushing the accelerators of the immune system. Jim and I independently discovered that the reactivation of the immune system by blocking two major negative regulators, CTLA4 and PD-1, can cure a significant portion of cancer patients.

## Results and discussion. Science as the prosecution of politics by other means?

**Fig 5:**
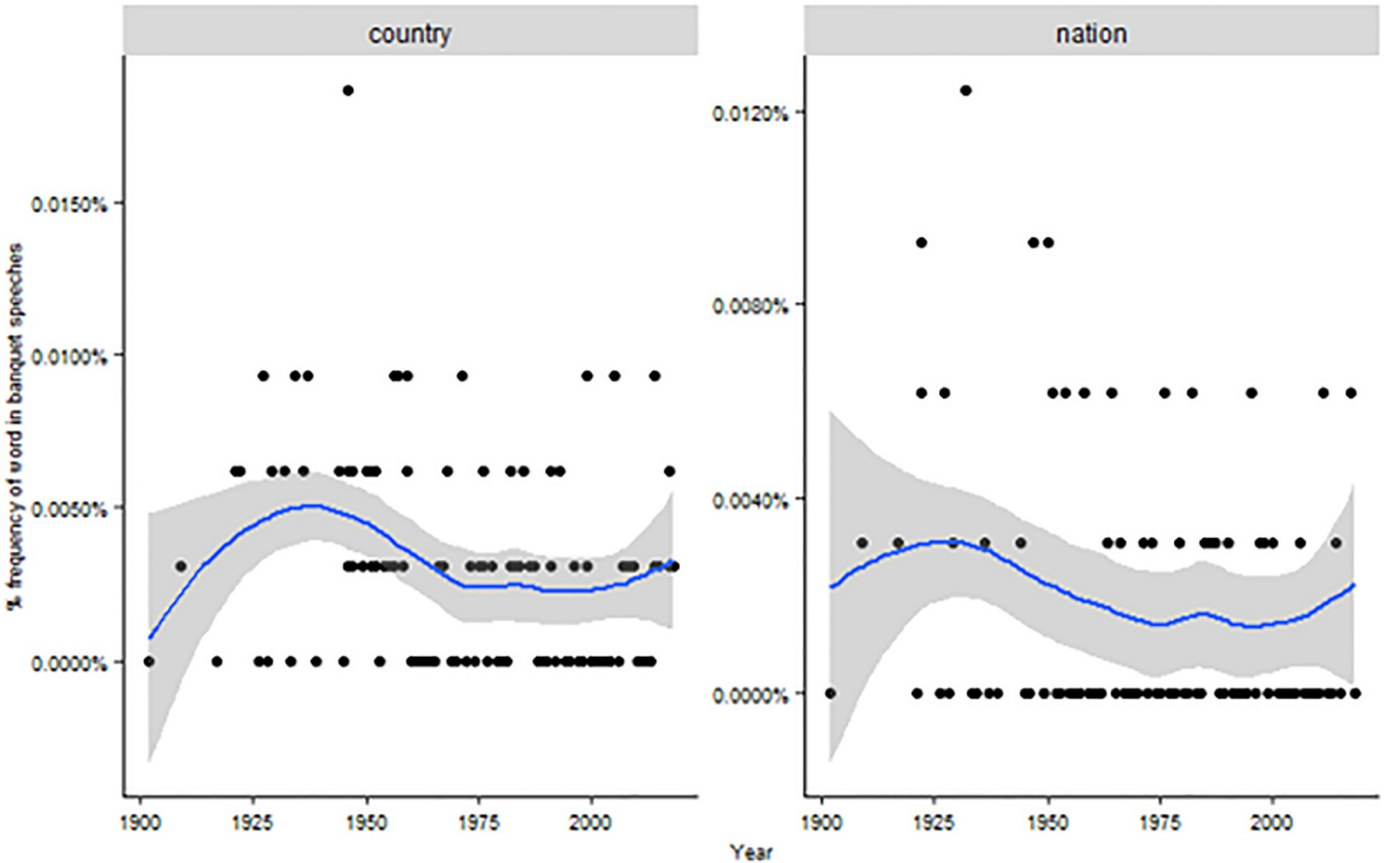
Mentions of “country” and “nation” across time. Dots represent the % of each term on the total of words for each year. The line represents the historical trend (confidence interval at 95%).

General, quantitative analysis of Nobel speeches may convey the impression of a strong focus on nationalism. “Country” and “nation” are frequently invoked by laureates, and especially the first term is more often used during the two World Wars (Fig 5).

However, a more in-depth analysis reveals that those terms are actually used to present the Nobel as a terrain where nationalistic stances and conflict among nations could find a context for peaceful competition and even cooperation [1, 2, 3, 20].

This tension between national pride and science internationalism is well exemplified by several speeches.

Herbert S. Gasser (Physiology or Medicine, 1944):

So high is the world-wide esteem of the judgments of the Nobel Committees, that there could not fail to be present a pardonable element of national rejoicing. It was something far different, however, that caused the occasion to be one never to be forgotten. Chords were struck in which there sounded in harmony sympathetic vibrations between your country and ours, resonant with our mutual love of learning, tolerance, freedom, and peace. And there were overtones of good will that caused the whole, in the ears of a war torn world, to seem to mount to a hymn to the international ideal to which Alfred Nobel devoted his life and his fortune.

The international character of science is interestingly reinforced through the association of the science Prizes with the other Prizes (Literature, Peace) established in the will of Alfred Nobel.

Irving Langmuir (1932, Chemistry):

He [Alfred Nobel] recognized the importance of science and of medicine as factors tending to increase human happiness. He regarded idealistic literature as a source of international good will and understanding. And he hoped that this good will would help lead to international peace; but wished also to stimulate direct constructive efforts toward peace between nations. This coupling together of science with international peace, is, I think, particularly significant. Science, almost from its beginnings, has been truly international in character. National prejudices disappear completely in the scientist’s search for truth.

Likewise, discourses about peace combine with the above-mentioned praise of the laureate’s own field and country.

Archibald Vivian Hill (Physiology or Medicine, 1922):

Physiology, I am glad to know, was the first science to forget the hatreds and follies of the War and to revive a truly international Congress: my own country, I am proud to boast, was happy to be its meeting-ground. For a while, my friend Meyerhof was an enemy: today he is again a colleague and a friend.

Thus, the Nobel Prize turns political enemies into colleagues, and scientific discussions overshadow political quarrels.

Archibald Vivian Hill (Physiology or Medicine, 1922):

Only three months ago, little suspecting the occasion of our next meeting, we were walking for several days together in the Donauthal, after another Congress, *discussing occasionally the neutralisation of the Rhine*, *but generally the neutralisation of acid in frog’s muscle*. We are glad to feel that your joint award to us, however unworthy we may know ourselves to be, is a seal of your approval, the approval of a people friendly to my nation and to his nation, of a brotherhood in Science between a German and an Englishman. Two things, therefore, are emphasised by our presence here today, the romance and adventure of discovery, and the brotherhood of Science (italics added).

After the Second World War and its tragic epilogue, a new dimension adds to the political discussion of science: its responsibility. In the words of laureates, science is increasingly described, rather than a positive force for mankind *per se*, as a force that has to be understood in all its potential implications and carefully used.

Edward Appleton (Physics, 1947):

It seems to me that we must recognize that the proper use of science is one of the most important challenges of the present day […] for the use that can be made of the same scientific knowledge can be good or it can be evil. It is only in the heart of mankind that the distinction can be made.

All the ambivalence of science’s social and political role, together with the ambivalence of the Prize itself and of the figure of its founder is dramatically captured by speeches like this by physicist Patrick Blackett (1948).

Now we find ourselves surrounded by rumours and threats of a third world war–a war which, if it comes, will be made more terrible than the last through the wonderful discoveries in atomic physics of the last decades. It is a curious comment on the unexpected twists of human history to note that Alfred Nobel used a fortune made through the invention and manufacture of explosives to endow most generously prizes for outstanding achievement in the arts of peace–and by so doing has stimulated and encouraged the great stream of discovery in pure physics, which culminated in the overwhelming devastation of Hiroshima. From this rostrum in previous years have spoken to you, as Nobel Laureates, nearly all the great scientists whose work has made the atomic bomb possible–the Curies, Rutherford, Bohr, Aston, Joliot, Lawrence, Hahn, to name only a few. Pure science has proved the most dangerous of pursuits. In the field of destruction, the wise words of J. J. Thomson are as valid as in the arts of peace: “Applied science makes improvements; pure science makes revolutions”.
